# Supplementary material for: Are carbon emissions trading and green financial instruments synergistic? -Comprehensive quantitative research based on content analysis
Source: PLoS One. 2024 Mar 7;19(3):e0298601. doi: 10.1371/journal.pone.0298601 (PMC10919601; doi:10.1371/journal.pone.0298601)
Supplement: S1 Data — (ZIP) [file pone.0298601.s001.zip › Data/地级市Z_F工作报告.zip!/╦─┤¿/░═╓╨╩╨/└┤╫╘╣1⁄2╓┌║┼í╛┬φ┐╦╩2╛▌═°í┐_2019.docx]

各位代表：

现在，我代表市人民政府，向大会报告工作，请予审查，并请市政协委员和其他列席人员提出意见。

一、2018年工作回顾

过去一年，面对错综复杂的经济形势和艰巨繁重的工作任务，在省委、省政府和市委坚强领导下，在市人大、市政协监督支持下，我们认真贯彻落实中央和省、市各项决策部署，大力实施“六大突破”、强化“六个推进”，真抓实干、开拓创新，较好完成了市四届人大二次会议确定的目标任务，推动川陕革命老区振兴发展示范区建设实现良好开局。

（一）经济发展稳中有进。全市完成地区生产总值645.88亿元、增长8.0%，全社会固定资产投资1127.01亿元、同口径增长9.2%，社会消费品零售总额321.45亿元、增长11.9%，城乡居民人均可支配收入分别增长8.9%和9.6%，金融贷款余额增长11.7%，居民消费价格涨幅控制在3.0%以内。投资保持稳定增长，281个市级重点项目完成投资784.3亿元、超时序进度1.2个百分点，民间投资增长4.1%；巴中恩阳机场如期建成，机场快速通道加快推进，巴陕高速全线通车，巴万高速加快建设，汉巴南城际铁路巴中至南充段可研批复前置要件全部完成，苍巴、镇广渝高速和米仓大道前期工作推进顺利，改造升级普通国省干线158公里、县乡公路272公里，建成建制村联网路1474公里、村内通组路4237公里；双桥水库完成蓄水验收，红鱼洞、黄石盘、湾潭河等10座在建水库和青峪口等15座水库前期工作加快推进，江家口水库可研通过水利部审查；城西220千伏等8个骨干输变电工程建成投运。社会消费提振有力，成功举办第一届秦巴山区绿色农林产业投资贸易洽谈会暨第二十七届中国西部商品交易会、第十六届光雾山红叶节、第四届“巴中云顶”茶文化旅游节，实现旅游收入248.83亿元、增长18.39%，旅游质量排名全省前5位；成功创建“巴山民宿”旅游公用品牌，新建“巴中枣林鱼”“南江黄羊大酒店”两大特色餐饮品牌直营店、加盟店20家，居然之家（巴中馆）等一批商贸服务企业投入营运。产业结构继续优化，三次产业结构由15.5:48.8:35.7调整为15.2:49.0:35.8；四大新型工业、四大特色农业产值分别占到工业、农业总产值的55.2%和18.7%，文旅产业对经济增长贡献率达到17.0%、提高1.2个百分点，科技进步贡献率达到50.5%、提高2个百分点。企业培育见到成效，“四百计划”深入实施，新增规上工业企业37户、建筑房地产企业107户、限上规上服务业企业192户、省级农业产业化龙头企业11户，国药天江等一批重点工业企业竣工投产，巴中经开区等4个工业园区被认定为省级新型工业化（特色）产业示范基地。县域经济加快发展，恩阳区、南江县入列全省县域经济发展先进县，恩阳区入列全省促进民营经济发展先进县和全省重大农村改革任务推进示范县，南江县入列全省农民增收工作先进县，平昌县入列全省“三农”工作先进县。一年的不懈努力，使全市经济不但保持了量的增长，而且有了质的提升，为实现经济高质量发展奠定了坚实基础！

（二）脱贫攻坚再创佳绩。减贫成效明显，全年实现7.46万贫困人口脱贫、168个贫困村退出，贫困发生率降至2.1%；巴州区、恩阳区正式摘帽并入列全省摘帽工作先进县，南江县摘帽达标工作有序推进，100个片区连片建设全面完成。贫困群众“两不愁、三保障”问题有效解决，发展脱贫产业37万亩，贫困家庭劳动力转移就业规模达9.6万人；易地扶贫搬迁9.2万人、提前两年完成“十三五”目标任务，改造农村危房2.78万户、土坯房5.26万户，解决9.65万贫困人口饮水安全问题，巩固提升94.7万人饮水安全，救助贫困中小学生8.8万人，义务教育阶段学生无因贫失学辍学，贫困群众全部参加基本医疗保险（含大病保险），全面推行医疗救助“一站式”服务，县域内住院费用自付比例控制在10%以内。合力攻坚态势良好，浙江丽水－巴中东西部扶贫协作、香港各界扶贫促进会定点帮扶南江县落地项目42个，到位资金1.5亿元；财政、信贷、社会投入扶贫资金近100亿元；土地扶贫流转城乡建设用地增减挂钩、耕地占补平衡指标1.6万亩，到位资金14.76亿元；在全省率先启动“四川扶贫”公益性集体商标使用试点；扶贫领域腐败和作风问题专项治理取得明显成效；巴山牧业董事长荣获全国脱贫攻坚“奋进奖”，“坚持‘五个融入’服务脱贫攻坚”的经验做法在全省推广。一年的风雨兼程，书写出决战贫困的胜利篇章，农村的日子越来越好，全市离全面小康的目标越来越近了！

（三）城乡建设有序推进。市中心城区建成区面积达到58平方公里、人口67万，城镇化率提高1.31个百分点、达到41.85%。城市面貌不断改善，启动市本级和三县城总规评估审视，中心城区控规及城市设计覆盖率达到100%；全市老旧小区新批增设电梯190部、居全省第1位，巴州城区完成祠堂街等3个特色街区打造，麻柳湾至巴河大桥滨河路全线贯通，新增城市道路22公里、滨河路健身步道3公里、绿化面积9万平方米、彩化面积3万平方米；兴文新区完成市规划馆改造提升，“一基地三中心”“市民之家”等业务用房建成投用；恩阳新城新增城市建成区面积1.4平方公里，平昌县获批成立省级经开区，南江县红塔新区全面建成，通江县高明新区加快建设。乡村振兴稳步推进，高质量承办全省乡村文化振兴发展现场会和全省秦巴山区“农村土坯房改造行动”现场会，巴州区和恩阳区入列全省城乡融合发展综合改革试验区，恩阳区和巴州区三江镇、平昌县土兴镇入列全省乡村振兴规划首批试点，四川乡村振兴学院在恩阳区成立；四大特色农业加快发展，提前一年完成粮食生产功能区和重要农产品生产保护区划定，粮食产量稳中有增，云顶、罗村茶入选“全国名特优新”农产品目录，南江县入列全国首批有机食品生产基地建设示范县，恩阳区荣获“中国芦笋之乡”称号；建成“产业园区+田园景区+新型社区”综合体11个、村史馆（农耕文明博物馆）35个，打造乡村旅游示范带5条、省级民宿达标户55户，通江县龙池村村规民约入选全国优秀村规民约；农村生活垃圾、污水处理率分别达到50%和30%，建成农村公厕125座。创建工作成效明显，光雾山－诺水河世界地质公园揭碑开园，巴中市入列全国旅游标准化试点市、全省红色旅游融合发展示范区，光雾山、王坪国家AAAAA级景区创建分别通过省检和省资源评价，新增国家水利风景区1个、历史文化名镇1个、全国最美森林小镇和省第二批特色小城镇各2个、“百镇建设行动”试点镇12个，新增第五批中国传统村落14个、全省“最美古村落”2个、全省第一批美丽渔村3个，建成幸福美丽新村260个、省级“四好村”110个、市级“四好村”263个。一年的奋力实干，进一步改善了城乡面貌，加速了城乡融合的进程，巴中大地变得更加多姿多彩！

（四）改革开放全面深化。全面创新改革试验41项年度改革任务全面完成，市政府签订市际、市厅、校地、院地等各类合作协议21份，招商引资到位资金553.24亿元。供给侧结构性改革取得实效，关停落后产能企业115家，淘汰燃煤锅炉44家；取消、降低和减免企业收费8项，实体经济企业成本降低1.5亿元。农业农村改革成效显著，农村土地承包经营权确权登记颁证全面完成，62个乡镇完成集体产权制度改革；巴中市入列全国农村集体产权制度改革试点市，通江县入列全国农村综合性改革试点县、全国农产品质量安全县、全国农村创业创新典型案例、四川省农村集体产权制度改革突出单位。“放管服”改革深入推进，取消行政权力事项12项、证明事项186项，一体化政务服务平台完成升级改造并实现市县乡全覆盖，“网上办”“马上办”“一次办”比例分别达到65%、56%和98%，工商登记实现“三十三证合一”，新登记市场主体增长20%。国资国企和投融资改革取得新成绩，组建市文旅集团、市交投集团、秦巴田园农业公司，设立川陕革命老区振兴发展基金，推动29家规上企业实施规范化公司制改制，南江黄羊交易中心产品成功上线，建成全国首个金融诚信教育基地，四板新增挂牌企业6家，资本市场实现直接融资39.53亿元。开放合作不断扩大，海军“巴中舰”建成入水，新增国内友城2个，成立巴商总会和4个异地巴中商会，8个项目（节会）入列川东北经济区“三个十大”推进方案，全年引进黄石旅游区等重大产业项目68个、到位资金86.23亿元。科技创新稳步推进，建成IT产业园4个、院士专家工作站5家、青年创业示范基地198家、省级科技企业孵化器2家、众创空间3家，新增国家高新技术企业7家、省级工程技术研究中心2家，新授权专利599件。一年的砥砺奋进，对内盘活了资源，对外拓展了发展空间，我们的发展变得更有动力、更有活力！

（五）民生福祉切实改善。全年一般公共预算民生支出占比72%，省十项民生工程及20件民生实事、市自选10件民生实事年度任务全面完成。教育和卫生健康事业加快发展，新开工建设城区学校6所，改造农村义务教育薄弱学校150所，建成乡镇标准化中心校30所，新建乡村教师周转宿舍1024套，化解义务教育阶段大班额587个，县域义务教育基本均衡发展目标全面实现，教育教学质量持续提升，高中试行“选课走班”，办理生源地助学贷款2.81亿元；组建城市医联体1个、县域医联体12个，新增异地就医全国联网即时结算地4个；新建村（社区）残疾人康复站120个、妇女之家和儿童之家200个、社区家长学校（家庭教育服务指导站）100个，开展新生儿疾病筛查1.8万余人次、妇女“两癌”筛查6万余人次，巴中市中医院通过三级甲等医院综合评审，通江县入列全省综合医改示范县，全市失能和半失能特困人员集中供养率达到30%。文体事业成果丰硕，成功举办第六届巴人文化艺术节，全国总工会文工团等赴巴演出精品剧目15场次，四川扬琴《守望》荣获中国曲艺“牡丹奖”，改革开放四十周年大型成果展获得社会广泛好评；实施广播“村村响”工程203个、电视“户户通”2万余户；成功承办第九届环中国自行车赛，新建农民体育健身工程152个，竞技体育成绩刷新历史纪录。社会治理持续加强，扫黑除恶专项斗争扎实推进，十大重点领域（行业）乱象和政府投资项目招投标突出问题得到有效治理，调解、信访、安全生产、社会治安管控、食品药品监管、防汛减灾和非洲猪瘟疫情防控等工作扎实有效，自然灾害实现“零伤亡”，安全生产事故数、死亡人数分别下降35.1%和36.1%，帮助农民工讨薪1.1亿元，社会保持和谐稳定。国防动员、双拥以及审计、统计、保密、档案、地方志、民族、宗教、外事、友协、侨务、对台、群团、关心下一代、社会科学、老龄、慈善、气象等事业和农民工权益维护与保障工作都取得了新成绩。一年的倾情付出，把温暖送进了百姓心里，把便利带到了群众身边，全市人民的获得感幸福感安全感明显增强了！

（六）生态环境持续向好。保持和巩固环境空气质量达标城市成果，城市环境空气质量优良率93.9%，城乡集中式饮用水水源水质达标率分别为100%、90%，国控出境断面地表水水质由III类提升至II类，森林覆盖率62.1%，土壤环境质量安全可控。污染防治有力有效，提请市人大常委会审议了《巴中市城乡污水处理条例（草案）》《巴中市扬尘污染防治条例（草案）》，中央、省环保督察及“回头看”反馈问题整改完成年度任务，完成县级饮用水源地环境问题整改和巴城大佛寺饮用水源地保护设施建设，化肥、农药使用负增长；关停“散乱污”畜禽养殖场36家，关闭拆除砖瓦窑80家，完成燃煤锅炉替代电量1355万度，单位GDP能耗下降2.5%；河（湖）长制全面落实，3754名河（湖）长开展巡河15.5万次。生态建设力度加大，启动28个矿山地质环境恢复治理，巩固退耕还林91.4万亩、新造林52万亩，新增森林面积22.6万亩，打造彩林廊道2000公里，延伸水系绿线217公里，空山国家森林公园和天马山国家森林公园分别入列中国森林体验基地、中国森林养生基地，诺水河省级自然保护区规划调整成功获批，南江县入列国家生态文明建设示范县和省级生态园林县城，恩阳区入列“绿水青山就是金山银山”实践创新基地。环境监管执法从严从实，严格环境准入，完成生态红线划定，完成重点排污企业在线监管设施安装并联网运行，开展“双随机”监管440余次，下达行政处罚决定150件，集中通报曝光环境违法典型案件9起，打击环境违法行为的高压态势持续形成。一年的持续攻坚，促进了人与自然的和谐共生，提升了全市的生态环境质量，巴中的天更蓝、水更绿、环境更美了！

各位代表！去年，我们在深入贯彻落实中央、省和市委决策部署的基础上，针对经济社会发展短板，持续实施了“八个一批”工作：一是推进一批滞后重点项目，22个停工半停工项目有序推进。二是放大一批基金效益，巴中川陕革命老区振兴发展产业基金和股权投资基金募集到位资金2.8亿元、完成投资1.35亿元，农业产业发展融资风险保障金为37家企业融资1.52亿元。三是引导一批民间投资，引进培育总部企业3家，引进民间投资项目251个，投资总额881.03亿元，开工建设239个。四是集聚一批金融产业，秦巴金融中心建成部分基础配套设施，巴中农商银行等4家金融机构启动迁建工作，新设立达州银行巴中支行、天府商品交易所南江黄羊交易中心、秦巴九鼎基金等金融机构7家。五是疏解一批老城城市功能，南池市场完成搬迁，市中心医院第二医疗区建成投运。六是集中整治一批城市不文明行为，全国文明城市创建实现良好开局。七是化解一批住建国土领域信访积案，旺福苑、坝中名苑等老难信访问题得到有序处理。八是打击一批违规违法行为，巴城的秩序日益规范。事非经过不知难。这“八个一批”都是“硬骨头”，在推进过程中，不少干部群众为此贡献了智慧、流下了汗水、耗费了心血。在此，我代表市人民政府向大家的无私付出表示衷心的感谢！

各位代表，这里有三个重点事项和一项经济指标需要作出说明：一是2018年市《政府工作报告》安排重点任务337项，已完成293项，按时间节点正常推进4项。其余40项工作中，北环线东段项目建设、光雾山国家AAAAA级景区创建、国家有机产品认证示范市创建等34项工作由于财政和环保政策调整、机构改革等原因，相关目标已按法定程序进行了优化；巴万高速清江至通江段项目建设、全社会固定资产投资等6项滞后工作已责成有关区（县）、市级部门在市政府全体（扩大）会上作出说明。二是区（县）政府和市级部门公开承诺办理的180件发展大事、民生实事，已办成171件，余下的巴中城镇污水处理设施、城乡垃圾处理设施PPP项目建设等9个事项按法定程序进行了调整，下一步将持续办理。三是我在人代会上承诺督办的5件民生实事已全面或超额完成，分别是：新建城区公共停车泊位10185个，新（改）建城乡公办幼儿园10所、城区公厕30座、城市书房9个、农贸市场53个。一项经济指标，即全市地方一般公共预算收入完成45.46亿元、下降0.15%，主要是国家减税降费、省减轻企业负担、市优化财政收入结构等三方面因素叠加影响的结果。

各位代表，过去一年能够取得这样的成绩，是省委省政府深切关怀、倾情帮助的结果，是市委正确决策、坚强领导的结果，是市人大和市政协严格监督、大力支持的结果，是全市广大干部群众艰苦奋斗、团结拼搏的结果。在此，我代表市人民政府，向全市人民，向人大代表、政协委员、各民主党派、工商联、无党派人士、各人民团体，向香港同胞、浙江丽水人民、友城兄弟以及所有关心支持巴中建设发展的社会各界人士，表示衷心的感谢！向驻巴部队、武警官兵、政法干警和全市综合性消防救援队伍、民兵预备役人员致以崇高的敬意！

各位代表，我们也清醒看到，全市经济社会发展还面临不少问题和挑战。从长远看，经济发展质量总体不高，产业基础薄弱、经济结构不优、市场机制不活、协调发展不足、开放程度不深、营商环境欠佳等问题比较突出。就当前而言，经济下行压力加大，实体经济运行成本较高，民间投资增长乏力；民生领域“短板”较多，脱贫攻坚任务艰巨；部分领域新旧矛盾叠加，社会治理面临新的挑战；政府职能转变不够到位，防范化解风险能力不足；少数公务人员庸政怠政，为民服务的质量和效率有待提高。对此，我们将高度重视，采取有力措施加以解决。也请各位代表和委员支持我们、帮助我们、监督我们。

二、2019年工作安排建议

2019年是新中国成立70周年，是深入贯彻党的十九大精神、决胜全面建成小康社会第一个百年奋斗目标的关键之年，做好各项工作意义重大。全市政府工作的总体要求是：以习近平新时代中国特色社会主义思想为指导，全面贯彻党的十九大、省委十一届三次全会和市委四届五次全会精神，认真落实中央、省委、市委经济工作会议和省委十一届四次全会、省政府第三次全体会、市委四届七次全会安排部署，坚持稳中求进工作总基调，坚持新发展理念，坚持推动高质量发展，坚持以供给侧结构性改革为主线，坚持深化市场化改革、扩大高水平开放，认真落实省委“一干多支、五区协同”“四向拓展、全域开放”战略部署，持续实施“六大突破”、强化“六个推进”，统筹推进稳增长、促改革、调结构、惠民生、防风险工作，保持经济持续健康发展和社会大局和谐稳定，推动川陕革命老区振兴发展示范区建设取得新的更大成效。

今年，全市经济社会发展主要预期目标是：地区生产总值增长7.8%，全社会固定资产投资增长8%，社会消费品零售总额增长12.5%，地方一般公共预算收入增长2.36%，城乡居民人均可支配收入分别增长9.0%、9.5%，城镇登记失业率、居民消费价格涨幅分别控制在4.4%和3.0%以内，环境保护和节能减排完成省下达目标任务。实现这些目标，重点要抓好以下工作：

（一）持续打好三大攻坚战。这是党中央确定的2020年前全党的重点工作，今年进入冲刺关键期，必须以踏石留印的作风、抓铁有痕的举措，夺取新的更大胜利。

坚决打好精准脱贫攻坚战。强化脱贫攻坚的政治责任感和历史使命感，全面落实《关于打赢脱贫攻坚战三年行动的实施意见》，坚持目标标准，下足“绣花”功夫，提升脱贫质量，实现南江县正式摘帽、通江县和平昌县摘帽达标、101个贫困村退出、6.09万贫困人口脱贫。聚焦“两不愁、三保障”，新（改）建特色种植业基地17万亩，贫困家庭劳动力转移就业规模达9.6万人，完成6704人易地扶贫搬迁计划，深入推进教育、健康、消费扶贫工作。建实用好脱贫攻坚项目库，统筹整合财政涉农资金21亿元，撬动金融投入75亿元，吸引社会投入10亿元。加大城乡建设用地增减挂钩和土地整理项目实施力度，积极推动耕地占补平衡全国统筹。扎实抓好浙江丽水－巴中东西部扶贫协作，推动香港各界扶贫促进会定点帮扶项目落地建设。强化“励志扶贫”和乡村文明治理，凝聚乡贤力量助推脱贫攻坚，新建贫困村文化室221个，深化扶贫领域腐败和作风问题专项治理。常态开展“回头看、回头帮”，实现持久稳定脱贫。

坚决打好污染防治攻坚战。持续抓好中央、省环保督察及“回头看”反馈问题整改，配合做好中央、省新一轮环保督察。全面打好蓝天保卫、碧水保卫、黑臭水体治理、巴河流域生态保护、饮用水水源地环境问题整治、环保基础设施建设、农业农村污染防治、“散乱污”企业整治等“八大战役”，持续扩大绿色生态优势。深入落实河（湖）长制，创新流域治理投融资机制，加快河域岸线划定和河道治理。扎实推进“九大绿化行动”，抓好退耕还林、湿地保护等生态工程，争取森林覆盖率达到62.5%。积极创建“国家气候标志”，建成巴中新一代天气雷达。大力推广绿色生产生活方式，抓好固废循环经济产业园建设，探索开展用能权有偿使用和交易制度试点。大力推进生态文明制度体系建设，完善环境保护多元投入机制，完成 “三线一单”编制和生态红线落地，健全企业排污许可、环境信用评价和网格化环境监管体系，推进环保地方立法工作，完善生态环境损害赔偿和责任追究制度，抓好国家生态文明建设示范市和“绿水青山就是金山银山”实践创新基地建设。

坚决打好防范化解重大风险攻坚战。深入落实防范化解重大风险9个专项方案，坚决守住不发生区域性系统性风险底线。严格政府债务管控，坚决遏制隐性债务增量，积极稳妥化解存量，支持融资平台公司实体化转型，加强债务审计和财政决算监督。强化金融风险防范，重点防控地方法人金融机构风险，健全市县两级地方金融监管体系，压实属地风险处置责任，加强重点风险金融机构派驻监督，严厉打击非法集资，切实整治金融乱象，抓好互联网金融风险防范。强化国土、住建领域风险防控，加大信访积案化解力度。

（二）积极扩大开放合作。开放赢得未来。要坚持对外开放不止步，抢抓“一带一路”“一干多支”发展战略机遇，加快融入全省区域发展新格局和立体全面开放新态势。

深化开放大通道建设。全力实施第三轮交通大会战，有序拓展巴中恩阳机场营运航线，推动巴万高速清江至通江段、巴广渝高速兴文互通、省道木涪路建成通车，开工建设汉巴南城际铁路巴中至南充段、苍巴高速、巴陕高速赤溪互通、米仓大道、北环线东段，力争镇广渝高速开工，加快推进南江、通江通用机场和广巴、巴达铁路扩能改造前期工作，启动秦巴大道延伸至清江段前期工作。新（改）建普通国省干线公路190公里，完成县乡道改造327公里、村道窄路加宽374公里，建成建制村联网路1210公里、村内通组路3700公里。抓好“十四五”交通运输规划前期研究工作，争取南江经苍溪至盐亭、通江经宣汉至开州高速纳入省高网规划。

深入拓展“四向”合作。主动融入川东北经济区协同发展，推动五市市际合作协议涉及巴中事项落地落实。促进成都巴中协同发展，新签订一批项目合作协议。积极参与川渝合作分工，推动与渝中区、沙坪坝区、西南大学的合作协议落地落实，谋划推进连接万州港前期工作。加强与泛秦巴区域的合作，联动抓好红色教育、文化传承、米仓古道申遗等工作。拓展与浙江丽水、香港的合作，积极融入长三角、粤港澳大湾区发展。扩大与湖北黄冈等国内友城的交流合作，推进国际友城建设。

务实抓好招商引资。建立招商项目策划包装“区县主体+行业主责+市投资促进部门统筹”工作机制，全年包装超亿元项目100个、超五亿元项目50个。精心制作四大特色农业、四大新型工业、四大现代服务业招商专案，建立健全在谈招商项目流程管理，完善市级领导挂联促进重大招商项目和重点企业制度，积极开展小分队招商，务实参加中国西部（四川）国际投资大会、中外知名企业四川行等各类重大平台活动，争取到位招商资金560亿元以上，引进重大产业项目不少于25个。

（三）推动经济高质量发展。结构不优是巴中经济的最大短板。要坚持从供给侧和需求侧两端发力，不断优化调整经济结构，提高发展的质量和效益。

深化供给侧结构性改革。依法依规淘汰造纸、砖瓦等落后或过剩产能，工业投资同比增长35%，完成技改投资60亿元以上、替代电量1100万度，促进传统产能转型升级。抓好产销对接，鼓励政府采购本地企业产品，推进企业入网、全民触网、电商示范、快递下乡，防范化解产品积压。健全房地产市场平稳健康发展长效机制，实施“一城一策、差别调控”，加强房地产市场供需双向调节，优化住房供应结构，增加高品质、改善性住房供给，确保市场稳定。贯彻银保监会新九条措施，支持企业依法参与市场化债转股，鼓励企业上市融资，提高资产证券化率。全面落实减税降费政策，降低制度性交易成本，强化企业用电、用气等优惠政策争取，抓好“路企”对接、推动铁路运输量价互保，进一步降低要素和物流成本。

持续扩大有效投资。抓实抓好基础设施等重点领域补短板三年行动，实施市级重点项目296个，完成年度投资776亿元。推动江家口水库、昭化－巴中500千伏双回线路工程、黄石旅游区等159个市级重点项目开工建设，完成投资466亿元以上。推动红鱼洞水库、黄石盘水库、四川好彩头食品生产基地等131个续建项目加快建设，完成投资310亿元以上。推动巴中西龛110千伏输变电新建、百顺中药等71个项目竣工投产达效。加快推进青峪口水库、青龙嘴水库等项目前期工作，新储备重点项目400个以上。

实施消费升级行动。推进全域质量强市示范城市、放心舒心消费城市创建，新（改）建农贸市场25个，组织企业开展产销对接、市场拓展活动300场次以上，吸引民间资本在北京、成都、重庆、广州等中心城市开设“巴中枣林鱼”“南江黄羊大酒店”特色餐饮品牌直营店或加盟店10家以上，鼓励“巴食巴适”在市外开设专卖店和旗舰店。支持容邦国际商业综合体、西部国际商贸城和祠堂街、麻柳湾等特色街区创新业态，增加体育、健康、家政等服务消费供给。推进农村区域性养老服务中心建设，发展“互联网+居家养老”服务，推动巴中农特产品电商平台销售，培育新兴消费增长点。

大力发展文旅经济。加快建设国家全域旅游示范区、全国旅游标准化试点市和全省红色旅游融合发展示范区，持续擦亮“山水、洞天、红色、人文、康养”五张金字招牌。扩大光雾山－诺水河世界地质公园品牌效应，加快光雾山、王坪国家AAAAA级景区创建工作，推进光雾和谷国际森林康养小镇建设，争取成功创建国家AAAAA级景区1个、国家AAAA级景区3个、省级以上森林康养基地6个，建成巴山民宿20家、森林人家20个，新（改）建景区厕所20座。引进“研学旅游”新业态，盘活现有旅游资源，持续优化提升旅游线路和丰富文旅产品，推进“六个一”文艺精品打造，新落地文旅产业项目5个，培育规上文旅产业单位5家，开发文化创意产品10件（套），办好第七届巴人文化艺术节、第十七届光雾山红叶节、第五届“巴中云顶”茶文化旅游节等节会活动，强化周边客源地及巴中首航城市旅游营销，力争全年综合旅游收入突破300亿元，文旅产业对GDP的贡献率达到18%。

加快培育现代产业。突破发展茶叶、核桃、道地药材、生态养殖四大特色农业，做大巴山牧业、秦巴田园等农业产业化龙头企业，推进国、省特色农产品优势区培育创建和全域有机产品认证示范区创建，做强巴中云顶、通江银耳、恩阳芦笋、南江黄羊、青峪猪等产业品牌，推进太极集团巴中中药材基地建设，新建省级以上现代农业园区1个、省级有机产品认证示范区1个，举办第二届秦巴山区绿色农林产业投资贸易洽谈会。大力发展食品饮料、生物医药、新能源新材料、电子信息四大新型工业，落实制造业强省战略，推进国家级经开区创建，加快白酒、食品、医药、茶叶、石墨等5大专业特色园区建设，实施“十亿级企业培育工程”，支持远鸿小角楼、江口醇争创全省白酒行业“新十朵金花”，推动国药天江达效扩产，加快建设石墨加工产业园和坪河原材料基地，争取规上工业增加值增长11.2%以上、四大新型工业产值占到规上工业总产值60%以上。突破发展旅游、康养、商贸物流、文化创意四大现代服务业，实施服务业发展“30工程”，加快建设恩阳空港物流园、秦巴金融中心，开工建设川东北中药材仓储物流基地，推动巴中农商银行总部迁建工程开工，争取省级金融机构为巴中未来五年授信2500亿元，新增信贷100亿元，引进（设立）各类金融机构3家，加强“险资入巴”工作，服务业中营利性服务业增加值增长9.0%以上、非营利性服务业增加值增长7.5%以上。

（四）促进民营经济健康发展。这是今年经济工作的重点任务。要聚焦政策、企业和环境深度发力，毫不动摇地鼓励、支持、引导、保护民营经济发展，充分激发市场活力。

推动政策全面落地。全面落实促进民营经济健康发展省“20条”、市“10条”政策措施，引导民营企业家坚定信心、安心发展。开展政府奖励扶持政策动态清理，做好“废、改、立”工作。建设全市民营企业政策信息发布平台，加强政策解读、宣传和培训工作，扩大政策知晓覆盖面。健全政策落实跟促机制，配套完善督查、考核、通报、约谈制度，实施落实效果第三方评估，坚决打通政策落地“最后一公里”。

营造一流营商环境。全面开展“营商环境提升年”行动，围绕构建“全省最优、全国一流”营商环境目标，进一步完善安商、亲商、优商措施，探索设立“企业宁静日”制度，全面落实市场准入负面清单和公平竞争审查制度，实现“法无禁止皆可为”。对接全省统一的企业维权服务平台，畅通民营企业诉求渠道，全面清理整治拖欠中小民营企业账款问题，依法保护企业家财产权、创新权和自主经营权。建立民间资本投资项目库，规范有序推进政府和社会资本合作。支持成立行业商（协）会和产业联盟，支持巴商总会建设，建立领导联系商（协）会制度和民营企业评议政府部门工作机制。强化诚信建设，努力构建“亲”“清”新型政商关系，坚决惩治损害营商环境的人和事，让“上顶、下压、中梗阻”者没有市场。

发展壮大民营企业。推进企业培育“四百计划”，年内净增规上工业企业30户、建筑房地产企业60户、限上规上服务业企业100户、省级农业产业化龙头企业30户。实施“市场主体增量提质行动”和“大企业大集团培育工程”，支持国企民企合作和民营企业跨地区跨行业整合，新注册小微工业企业100户，新培育“小巨人”“成长型”中小企业100户、“两化”融合示范企业10家。支持民营企业向资本市场进军，争取1－3家企业在新三板或主板上市。畅通政银企沟通渠道，引导金融机构加大对民营和小微企业的信贷支持力度，深入破解融资难融资贵问题。鼓励民营企业建立现代企业制度，抓好企业家队伍、人才培育和企业文化建设。

（五）推进全面改革创新。“唯改革者进”。做好今年各项工作，必须坚持向改革要动力，进一步解放思想、爬坡过坎，全面完成各项改革年度任务。

深化重点领域改革。推进川陕革命老区综合改革试验，争取在生态补偿、差别化财政政策、资源开发体制等方面先行先试。全面完成政府机构改革，实现职能、方式、作风新转变。健全财政预算绩效管理制度体系，推动预算和绩效一体化管理。持续推进社会保险费和非税收入征管职责划转。优化国有资本布局，集中培育市文旅集团、市交投集团等骨干企业，加快公用事业板块资产整合，使之成为重大基础设施、重大产业和重大社会事业项目的投资主体、融资载体、营运实体。深入落实省国资委与巴中签订的战略合作协议，全方位多层次拓展投融资空间，大力推动省属国企入巴。加快推进川陕革命老区振兴发展基金募投、力争基金投资额达到10亿元，完成1－3家国有企业上市前期培育工作。

推进基础性改革。建立健全医疗机构党委领导下的院长负责制，全面启动公立医院薪酬制度改革和取消耗材加成改革，实施教育体制机制和新时代教师队伍建设改革，推进普通高考综合改革，抓好统计改革和审计机关管理体制改革，推进水电气等资源价格改革，持续深化供销合作社综合改革，启动普通国道市场化养护改革。

抓好“双创”工作。开展新一轮科技合作市厅会商，推进与哈尔滨工业大学、中科院金属研究所等校（院）地合作，抓好国家农业科技园区提升和验收，主动对接国、省科技成果转移转化示范区，新增国家高新技术企业5家以上，备案国家科技型中小企业50家以上、院士专家工作站3家以上，引进转化科技成果30项，申请专利不少于1300件，实现高新技术企业主营业务收入110亿元。深入实施全民创新创业“四大行动”，全面落实省加强农民工服务保障16条措施和推进返乡下乡创业22条措施、巴中市保障农民工工资支付20条规定，抓好农民工法律援助和维权服务，打好“治欠保支”攻坚战。

（六）统筹推进城乡和区域协调发展。这涉及到当前我们面临的两大突出矛盾。要以实施乡村振兴战略和新型城镇化为契机，大力发展县域经济，促进城乡公共服务均等化，加快解决发展不协调不充分问题。

深入实施乡村振兴战略。编制市、区（县）和重点示范乡镇乡村振兴五年规划，高质量完成恩阳区和巴州区三江镇、平昌县土兴镇乡村振兴试点。加大农村改革力度，深入推进农房一体登记改革，探索农村宅基地“三权分置”改革，完成79个乡镇集体产权制度改革。加快建设“美丽巴中·宜居乡村”，推进农村人居环境整治三年行动，实施农村污水处理、农村生活垃圾治理、农村村庄治理、农村“厕所革命”、畜禽粪污治理和资源化利用五个专项行动，抓好170个村新村配套设施建设，农村生活垃圾、污水处理率分别提高20个百分点，新（改）建农村公厕212座、农村户用厕所3.1万座，建成 “三区同建”点位8个、中心村21个、聚居点100个、幸福美丽新村200个、村史馆（农耕文明博物馆）36个。培育认定农业职业经理人300名，回引2000名农民工返乡下乡创业。

加快推进新型城镇化。实施城市高质量发展行动，划定“三区三线”，积极做好国土空间规划编制前期工作，持续推进城市“双修”，加快开展“两拆一增”，有序实施新能源汽车充电桩、停车场和农贸市场规划，加快建设城市道路、地下管廊、污水处理设施、绿地公园等市政项目，市中心城区新增城市绿化面积5万平方米、彩化面积2万平方米。推动巴州津桥湖、檬子河、龙泉片区有序开发，加大老城功能疏解力度，持续补齐城市功能短板；大力推进兴文新区开发建设，推动市法院、市检察院、市公安局等业务用房建成投用，抓好市第一人民医院、市骨科医院、市精神病康复医院、巴中粮食现代物流园区等重点项目建设，加快建设产业新城、人居新城；完善恩阳新城基础设施和公共服务配套，推进义阳大桥、义阳山南路、恩阳区华西附属第二医院分院等重点项目，加快建设产业新城、旅游新城、空港新城。实施“小县优城”行动，深化南江县国家新型城镇化试点、公园城市建设试点和通江县全国结合新型城镇化开展支持农民工等人员返乡创业试点、平昌县宜居县城建设试点，大力支持平昌省级经济开发区建设。持续抓好全国文明城市创建，继续推进9个国家级重点镇和24个省级试点镇建设，培育创建市级特色镇13个、特色小镇6个，争创全省特色小城镇2个。推进农业转移人口市民化，全市城镇化率提高1.2个百分点。

做大做强县域经济。实施特色经济强县行动，支持区（县）有序争创全省生态建设标杆县、工业强县、农业强县、服务业强县、信息化强县、天府旅游名县，积极创建全省县域经济发展强县、先进县和进步县。深化扩权强县改革，完善扩权县管理体制和财税分享机制，赋予县级更大自主权，支持符合条件的县开展撤县改市前期工作。完善县域经济发展考核办法，加强激励约束，促进县域经济质量、总量“双突破”。

（七）保障改善民生和加强社会治理。民生连着民心。我们要坚持促发展与惠民生并重，加快社会领域公共服务补短板强弱项提质量，牢牢守住社会稳定底线。

着力办好民生实事。加大基本民生保障投入，确保民生支出不低于一般公共预算支出的72%。全面办好省下达30件民生实事，扎实抓好市自选10件民生实事。坚持就业优先，统筹抓好高校毕业生、农民工、退役军人等群体就业工作，支持企业稳定就业岗位，力争城镇新增就业2.8万人以上。深入实施全民参保计划，参保人数争取达到550万人次，市、县、中心镇社保自助服务覆盖面达到80%以上。完善医疗保障、社会救助、社会福利、慈善公益等保障制度，妥善解决城乡贫困群众困难。改造城镇危旧房棚户区4100套、农村危房2.1万户、农村土坯房5.6万户，实施电梯增设三年计划，除险加固病险水库100座。

大力发展教育和卫生健康事业。优先发展教育事业，加强教师队伍建设，持续培育“名师名校”，引领全市教育高质量发展。务实推进课后服务，切实解决中小学生课外负担重、择校热等突出问题，化解义务教育阶段大班额230个，争取学前教育普惠性幼儿园学位占比达到75%，统筹规划城区高中教育阶段学校建设工作，完善高考综合改革基础保障条件，推进职业教育产教融合、校企合作，强化平安校园建设投入保障。实施改善医疗服务三年行动计划，持续培育“名医名科名院”，建好做实医联体和医共体，大力推动中医药事业发展，加快推进巴州区第二人民医院老年病院区等项目，广泛开展爱国卫生运动，推进健康村镇健康细胞工程建设，抓好重大疾病综合防治，加强医德医风建设和乡村医生管理，依法惩治医闹行为。切实保障妇女儿童、老人、残疾人等合法权益，深化公办养老机构改造提升和养老院服务质量建设专项行动，大力加强城乡养老服务体系建设，实施好“五助一帮”“童伴计划”、妇女“两癌”免费筛查和残疾儿童康复救助等公益项目，新建城乡社区儿童之家150个、残疾人村（社区）康复站120个。

加快发展文化体育事业。推进文化繁荣“四大工程”，常态开展“三下乡”“精品剧目进巴中”“律动巴山”等文化惠民、展演活动，设立大巴山文艺奖。推进米仓古道申遗，启动革命文物资源名录编制，加快红军石刻标语群保护规划编制，推进南龛摩崖造像抢救保护工程。加快市体育中心、市老干部活动中心、望王山国际山地户外运动示范基地建设，推进全民健身，新建农民体育健身工程400个，办好第五届市运会。推进基层“扫黄打非”示范网格创建和网格化管理。

全面加强社会治理。健全社会治安防控体系，纵深推进扫黑除恶专项斗争，深化十大重点领域（行业）乱象和招投标、惠农惠民补贴“一卡通”、非法营运等专项整治，依法打击各类违法犯罪，抓好反分维稳、反恐防暴、反邪防邪、禁毒斗争、防范和打击电信诈骗等重点工作。强化重点行业领域安全生产和食品药品监管，抓好动物疫病防治，坚决守护生命安全和“舌尖上的安全”；健全应急管理体制机制，抓好防汛抗旱、极端天气应对、地质灾害防治和水文气象预报工作；加强综合性消防救援队伍和公共消防设施建设，强化火灾防控和消防安全隐患整治，挂牌督办重大火灾隐患，加快推进国家安全发展示范城市建设。加强国家安全教育，深入实施“七五”普法规划，深化公共法律服务体系建设，坚持发展新时代“枫桥经验”，扎实做好群众信访工作，加强城乡社区治理。

大力支持工会、共青团、妇联、侨联、残联、科协、关工委、工商联等开展工作，加快发展老龄、残疾人等事业，扎实抓好档案、地方志、统计、民族、宗教、外事、友协、侨务、对台、保密、地震、人防、保险等工作，做好第四次全国经济普查和第三次全国土地调查，积极推进军民融合发展，启动新一轮全国双拥模范城创建，大力支持国防和军队建设，抓好国防动员、退役军人服务管理、优抚优待等工作。

各位代表！伟大的事业从来都不是一蹴而就的，需要我们一点一滴去积累，一步一步去完成。上面我报告的7个方面24项具体工作，希望大家大力支持、积极宣传、主动参与，在市委坚强领导下，凝聚全市人民同心同德、真抓实干，抓细抓实抓到底，积小成为大业，共同绘就美好蓝图！

三、持续加强政府自身建设

“打铁还须自身硬”。我们将坚定扛起建设川陕革命老区振兴发展示范区的历史责任，强化推动巴中后发赶超、振兴发展的使命担当，主动适应新形势、顺应新期待，围绕“五个第一”导向，持续加强政府系统党的政治、思想、组织、作风、纪律建设，不断提升政治理论和业务水平，努力建设人民满意的服务型政府。

持续加强政府系统党的政治建设。旗帜鲜明讲政治，增强“四个意识”、坚定“四个自信”、做到“两个维护”，自觉在思想上政治上行动上同以习近平同志为核心的党中央保持高度一致，坚决推动中央大政方针和省委省政府、市委决策部署落地见效。认真落实全面从严治党主体责任，严明政治纪律和政治规矩，严肃党内政治生活。扎实开展“不忘初心、牢记使命”主题教育，永葆忠诚、干净、担当的政治本色。

持续加强法治政府建设。坚持依法行政，自觉接受人大及其常委会的法律监督、工作监督和政协民主监督，严格落实人大讨论决定重大事项、政府重大决策出台前向人大报告等制度，认真办理人大代表建议和政协委员提案，广泛听取民主党派、工商联、无党派人士和社会各界的意见。支持开展监察和司法监督，主动接受社会和舆论监督。积极推动重点领域立法，抓紧开展烟花爆竹燃放、文明行为管理等立法调研和起草工作，推动出台《巴中市查处非法出租汽车客运若干规定》，配合市人大常委会做好城乡规划、城乡污水处理、扬尘污染防治等相关立法工作，推进行政执法与刑事司法衔接，进一步提升地方治理法治化水平。

持续加强创新政府建设。加快“最多跑一次”改革，用好一体化网上政务服务平台，完善“市民之家”功能，年内实现80%的事项“全程网办”、100%的事项“最多跑一次”、企业和群众“只进一道门，办成所有事”。推进公共资源交易监管体制机制创新和全流程电子化，常态开展工程建设项目异地远程评标，探索推行市内政府采购异地远程评标。推进政策制定和执行创新，强化新型智库建设，突出政策差异化和协调性，鼓励创造性贯彻落实政策，不搞“一刀切”“一锅煮”。健全正向激励、容错纠错、关心关爱机制，大力营造尊重劳动尊重创造的工作氛围、诚信和谐的人际关系环境和理解宽容的干事创业环境，努力让流汗流血的人不流泪。

持续加强民生政府建设。切实履行政府民生责任担当，继续实施“八个一批”，推动区（县）政府和市政府工作部门公开承诺办理一批发展大事和民生实事。健全民生工作责任体系，全面落实粮食安全责任制、“菜篮子”市长负责制、促进农民增收责任制，实行定期通报和责任约谈制度，促进民生工作责任层层落实。加大民生工作“阳光问政”力度，用好“三信两直播一热线”，及时回应社会关切，在政府和群众、社会间架起一道道永不阻断的桥梁。

持续加强廉洁政府建设。认真落实党风廉政建设责任制，深入落实《准则》和《条例》，严格执行《贯彻落实中央八项规定、省委省政府十项规定精神和市委市政府九项规定实施细则》，大力整治形式主义和官僚主义，持续培育精文简会、务实调研、亲力亲为、立说立行四项新风。加快建设节约型政府，牢固树立“过紧日子”思想，各级政府机关一律按照不低于5%的幅度压减一般性支出。强化廉政风险防控措施，抓好巡视巡察反馈问题整改，依法从严开展审计，管好用好公共资金、公共资源和国有资产。

各位代表！经广泛征求意见，今年市政府主要负责同志亲自督办事项共计5件，分别是：新建城乡公办幼儿园5所，新（改）建A级旅游景区、度假区、生态旅游示范区旅游厕所20座，年底前市中心城区全面实现餐厨剩余物集中统一收运和减量化、资源化、无害化处置，新建“菜篮子”标准园5个、蔬菜基地1万亩，市中心城区增设10处市民休憩设施。

各位代表！时代竞风流，盛世谱华章。今年是本届政府履职第三年，也是实施“十三五”规划承上启下的关键年，任务繁重，使命光荣。让我们更加紧密地团结在以习近平同志为核心的党中央周围，在市委坚强领导下，锐意进取、克难攻坚，为加快建设川陕革命老区振兴发展示范区作出新的贡献，以优异成绩迎接新中国成立70周年！
